# Efficacy of Ceftazidime and Cefepime in the Management of COVID-19 Patients: Single Center Report from Egypt

**DOI:** 10.3390/antibiotics10111278

**Published:** 2021-10-20

**Authors:** Ragaey A. Eid, Marwa O. Elgendy, Ahmed O. El-Gendy, Sara O. Elgendy, Lassaad Belbahri, Ahmed M. Sayed, Mostafa E. Rateb

**Affiliations:** 1 Department of Tropical Medicine, Faculty of Medicine, Beni-Suef University, Beni-Suef 62521, Egypt; ragaeyahmad@med.bsu.edu.eg; 2 Department of Clinical Pharmacy, Teaching Hospital of Faculty of Medicine, Faculty of Medicine, Beni-Suef University, Beni-Suef 62514, Egypt; Marwa.elgendy@nub.edu.eg; 3 Department of Clinical Pharmacy, Faculty of Pharmacy, Nahda University (NUB), Beni-Suef 62513, Egypt; 4 Department of Microbiology and Immunology, Faculty of Pharmacy, Beni-Suef University, Beni-Suef 62513, Egypt; Ahmed.elgendy@pharm.bsu.edu.eg; 5 Department of Clinical and Chemical Pathology, Faculty of Medicine, Beni-Suef University, Beni-Suef 62521, Egypt; drsaraosama88@med.bns.edu.eg; 6 Laboratory of Soil Biology, University of Neuchatel, 2000 Neuchatel, Switzerland; lassaad.belbahri@unine.ch; 7 Department of Pharmacognosy, Faculty of Pharmacy, Nahda University, Beni-Suef 62513, Egypt; 8 Department of Pharmacognosy, Faculty of Pharmacy, AlMaaqal University, Basra 61014, Iraq; 9 School of Computing, Engineering & Physical Sciences, University of the West of Scotland, Paisley PA1 2BE, UK

**Keywords:** ceftazidime, cefepime, SARS CoV-2, M^Pro^, COVID-19, in silico, glucocorticoids

## Abstract

The purpose of this study was to explore the value of using cefepime and ceftazidime in treating patients with COVID-19. A total of 370 (162 males) patients, with RT-PCR-confirmed cases of COVID-19, were included in the study. Out of them, 260 patients were treated with cefepime or ceftazidime, with the addition of steroids to the treatment. Patients were divided into three groups: Group 1: patients treated with cefepime (124 patients); Group 2: patients treated with ceftazidime (136 patients); Group 3 (control group): patients treated according to the WHO guidelines and the Egyptian COVID-19 management protocol (110 patients)/ Each group was classified into three age groups: 18–30, 31–60, and >60 years. The dose of either cefepime or ceftazidime was 1000 mg twice daily for five days. Eight milligrams of dexamethasone were used as the steroidal drug. Careful follow-ups for the patients were carried out. In vitro and in silico M^pro^ enzyme assays were performed to investigate the antiviral potential of both antibiotics. The mean recovery time for Group 1 was 12 days, for Group 2 was 13 days, and for Group 3 (control) was 19 days. No deaths were recorded, and all patients were recovered without any complications. For Group 1, the recovery time was 10, 12, and 16 days for the age groups 18–30, 30–60, and >60 years, respectively. For Group 2, the recovery time was 11, 13, and 15 days for the age groups 18–30, 30–60, and >60 years, respectively. For Group 3 (control), the recovery time was 15, 16, and 17 days for the age groups 18–30, 30–60, and >60 years, respectively. Both ceftazidime and cefepime showed very good inhibitory activity towards SARS CoV-2′s M^pro^, with IC_50_ values of 1.81 µM and 8.53 µM, respectively. In conclusion, ceftazidime and cefepime are efficient for the management of moderate and severe cases of COVID-19 due to their potential anti-SARS CoV-2 activity and low side effects, and, hence, the currently used complex multidrug treatment protocol can be replaced by the simpler one proposed in this study.

## 1. Introduction

**Figure 1:**
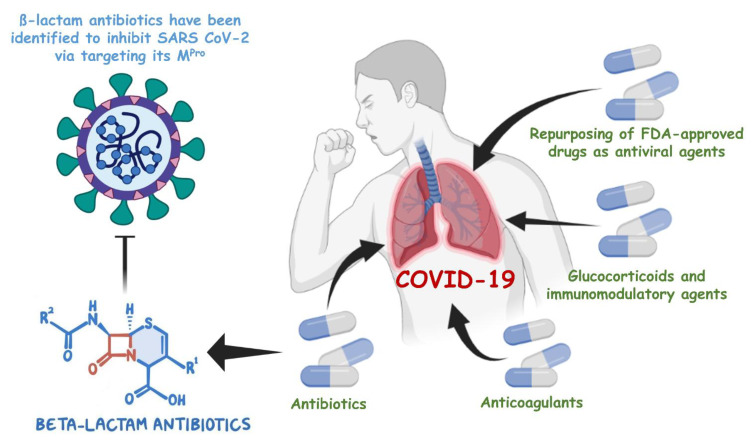
Schematic representation of the currently used therapeutics against COVID-19.

The whole world is confronting the COVID-19 (SARS CoV-2) pandemic. This virus is highly infectious, with a high morbidity rate [1]. Currently, there are no specific treatments for COVID-19, and the globally applied therapeutic strategies are based on repurposing the available drugs as antiviral agents (e.g., ivermectin and niclosamide) [2,3,4,5,6] or controlling the host immune response by using immunomodulatory agents and corticosteroids [7,8]. Moreover, anticoagulants were reported to be used in patients with severe COVID-19 illness [9,10]. Besides, antibiotics have been used extensively as a protective measure against secondary bacterial co-infection or superinfections [11,12] (Figure 1). Although the use of antibiotics with COVID-19 patients is still controversial, they have shown considerable therapeutic benefits, particularly with patients with severe pneumonia or those receiving glucocorticoids or other immunosuppressant therapeutics [13,14,15,16]. Another major concern of the uncontrolled use of antibiotics is the subsequent rapid development of resistance [17,18]. Accordingly, using a limited number of already available antibiotics that can also exert antiviral activity against SARS CoV-2 will have great potential in improving the recovery rate of COVID-19 patients and slowing down the emergence of antibiotics resistance.

Recently, third-generation cephalosporin antibiotics have shown considerable in vitro anti-SARS CoV-2 activity, with IC_50_ values ranging from 12–46 µM [19]. Another recent study has shown that some ß-lactam antibiotics can inhibit the SARS CoV-2 main protease (M^Pro^) (i.e., one of the key viral targets) [20]. Accordingly, we decided to initiate an observational study to evaluate the outcomes of using dexamethasone with broad-spectrum cephalosporine antibiotics in COVID-19 patients with moderate or severe illness. In this study, ceftazidime as third-generation and cefepime as a fourth-generation cephalosporin antibiotic were selected. These cephalosporin antibiotics have frequently been utilized as empirical antibiotic therapy in nosocomial infections with a reduced association of bacterial resistance. Moreover, ceftazidime has recently shown in vitro inhibitory activity against SARS CoV-2 (IC_50_ = 46.14 µM); however, its exact mode of action is yet to be discovered [19]. Based on the recent findings of Malla and co-workers [20], the ß-lactam scaffold of ceftazidime may enable it to target the viral M^Pro^. From a chemical point of view, cefepime is structurally close to ceftazidime. Hence, it has an outstanding potential to inhibit SARS CoV-2 by targeting the same enzyme (i.e., M^Pro^). Consequently, in addition to evaluating the therapeutic benefits of both antibiotics in COVID-19 patients, we also aimed, in this investigation, to assess their SARS CoV-2 M^Pro^ inhibitory activity.

## 2. Materials and Methods

A randomized study was conducted by including 370 (162 males) patients with reverse transcription-polymerase chain reaction (RT-PCR)-confirmed COVID-19. All of the participants fulfilled the inclusion criteria. The Research Ethical Committee of the Faculty of Pharmacy at Beni-Suef University authorized the study protocol (REC-H-PhBSU-19004) in accordance with the Declaration of Helsinki. Participants provided written informed consent. The clinical study was held in the isolation department of Beni-Suef University Hospital from 15 March 2021 to 20 May 2021.

### 2.1. Inclusion Criteria

- Patients within an age group of more than 18 years;
- Positive cases of COVID-19 by RT-PCR test;
- Moderate or severe cases with typical symptoms;
- Patients who were treated according to the national standard treatment protocol.

### 2.2. Exclusion Criteria

- Patients with severe hepatic diseases;
- Pregnant and lactating patients;
- Patients within an age group of less than 18 years.

### 2.3. Procedure

Only moderate and severe cases that were diagnosed according to the WHO ((https://www.who.int/publications/i/item/WHO-2019-nCoV-clinical-2021-1), (accessed on 20 September 2021). and Egyptian Ministry of Health [21] diagnosis criteria were considered in this study. The patients were randomly treated with cefepime or ceftazidime with the addition of steroids to the treatment protocol. The dose of cefepime was 1000 mg twice daily for five days, and the dose of ceftazidime was 1000 mg three times daily for five days. The steroidal drug that was used was 8 mg of dexamethasone. The patients were divided into two groups: Group 1: patients treated with cefepime (124 patients); Group 2: patients treated with ceftazidime (136 patients); Group 3 (positive control group): patients treated according to the WHO guidelines (https://www.who.int/publications/i/item/WHO-2019-nCoV-clinical-2021-1), (accessed on 20 September 2021). and the Egyptian COVID-19 management protocol [21], neither of which includes cefepime or ceftazidime (110 patients). The protocol of treatment used for Group 3 included the following therapeutics: (i) supportive multivitamins; (ii) favipiravir, remdesivir, hydroxyl chloroquine, and ivermectin as antiviral agents; (iii) anticoagulants according to the D-dimer level; (iv) colchicine and dexamethasone as anti-inflammatory and immunomodulatory agents.

The patients were followed up daily until the improvement in disease symptoms. In addition, a follow-up was accomplished regarding the duration of steroid utilization, which was the whole treatment period (recovery time). The recovery time (treatment duration) is calculated as days from positive confirmed COVID-19 PCR to the resolution of the symptoms. To detect the effect of age on the recovery time from the disease, each group was classified into three age groups: 18–30, 31–60, and more than 60 years.

### 2.4. In Vitro M^Pro^ Inhibition Assay

M^Pro^ inhibition assay was carried out according to the protocol of the commercially available assay kit (i.e., SARS CoV-2 M^Pro^ assay kit; Catalog #: 79955-1, BPS Bioscience, Inc., Allentown, PA, USA). The covalent inhibitor, GC376, was utilized as a reference inhibitor (IC_50_ = 0.22 µM.). The main principle of this assay was to produce fluorescence (observed at 460 nm for emission and 360 nm for excitation and excitation) upon the substrate cleavage by the viral M^Pro^. To prepare for the enzyme reaction, 10 µL of each test compound at varying concentrations were placed into a 96-well plate followed by adding 30 µL of a diluted protease solution (15 µg/mL). The reaction mixture was allowed to stand for 30 min at room temperature. Subsequently, 10 µL of the substrate was added to the reaction buffer to be dissolved and finally added to the reaction mixture to reach a final concentration of 40 µM. Finally, this reaction mixture was incubated for 4 h at 20 °C, and the produced fluorescence was measured by TECAN spark microplate-reading fluorimeter.

### 2.5. In Silico Investigation

#### 2.5.1. Ensemble Docking

All molecular docking experiments were carried out using AutoDock Vina software [22]. The M^Pro^ crystal structure of PDB code: 6LU7 [23] was prepared and then used for docking experiments. The active site for docking was located according to the enzyme’s co-crystallized ligand (i.e., a grid box was added to enclose the co-crystalized ligand, and hence the active site required for docking). The co-ordinates of the active site used for docking were set to be: x = −12, y = 12.5, z = 67, and the size of the searching box was set to be 10 Å. M^Pro^’s active site has been reported to be relatively flexible, and thus we used molecular dynamic simulation-derived conformers sampled every 10 ns over a total simulation time of 100 ns (a total of 10 conformers used for docking) for docking in order to account for this flexibility (i.e., ensemble docking) [19,24]. Thereafter, the average of these docking experiments was taken for each compound. Finally, the resulting docking poses were ranked according to their binding energy scores and then visualized and analyzed using Pymol software [22].

#### 2.5.2. Molecular Dynamic Simulation

Desmond v. 2.2 software was used for performing MDS experiments [25,26,27]. This software applies the OPLS force field. Protein systems were built via the System Builder option, where they were embedded inside a box of water (i.e., TIP3P) alongside 0.15 M of both Na^+^ and Cl^−^ ions. Subsequently, this prepared system was allowed to be energy-minimized and equilibrated for 15 ns. Ligand parametrizations were carried out by the software automatically according to OPLS force field during the system preparation step. For MDS carried out by NAMD software [28,29], ligand parametrizations were carried out according to CHARMM27 force field using the VMD plugin Force Field Toolkit (ffTK).

The free energy perturbation (FEP) method was used for calculating binding free energies (ΔGs). First, the input files and the applied script were prepared using the online application: CHARMM-GUI Free Energy Calculator (http://www.charmm-gui.org/input/fec) (accessed on 20 September 2021). [29]. Second, these inputs were loaded into NAMD to perform the dynamic simulation-based calculations. For each ligand, 20 ns of FEP dynamic simulations were carried out, where the final 5 ns were used for measuring free energy values [28,29]. The MDS-derived trajectories were analyzed and visualized by VMD software (University of Illinois, Champaign, IL, USA) [30].

## 3. Results

### 3.1. Clinical Outcomes

In this study, the patients, consisting of 162 males and 208 females, were monitored from 15 March 2021 to 20 May 2021 in Egypt. The oldest patient was 85 years, and the youngest patient was 18 years. A total of 24% of the patients suffered from diarrhea, 21% from anosmia, 23% from dysgeusia, 15% from chest pain, 17% from sore throat, 22% from vomiting, 21% from dizziness, 19% from fatigue, 43% from bony pain, 51% from cough, 10% from skin rash, and 15% suffered from rhinorrhea.

The mean steroid duration used in Group 1 was 5.07 ± 0.25 days, for Group 2, it was 5.66 ± 0.28 days, and for Group 3 (control), it was 7.66 ± 0.25 days. Moreover, the mean treatment duration (recovery time) for Group 1 was 12.33 ± 0.67 days, for Group 2, it was 13.29 ± 0.62 days, and for Group 3 (control), it was 18.66 ± 0.56 days. All of the patients recovered at the end of the study and did not need mechanical ventilation.

For Group 1, the mean recovery time was 10 ± 0.32, 12 ± 0.36, and 16.3 ± 0.57 days for the age groups: (i) 18–30 years, (ii) 30–60 years, and (iii) more than 60 years, respectively. For Group 2, the recovery time was 11.5 ± 0.27, 13.2 ± 0.64, and 15 ± 0.65 days for the age groups: (i) 18–30, (ii) 30–60, and (iii) more than 60 years, respectively. For Group 3 (positive control), the recovery time was 14.75 ± 0.55, 15.9 ± 0.32, and 17.3 ± 0.29 days for the age groups: (i) 18–30, (ii) 30–60, and (iii) more than 60 years, respectively.

The previous results indicated that the use of either ceftazidime or cefepime, along with steroids, for the treatment of moderate and severe cases of COVID-19 led to recovery times comparable with those resulting from treatment with standard of care therapeutics (i.e., positive control). Accordingly, patients will benefit well from our proposed simple protocol, which consists of only two drugs (dexamethasone + either ceftazidime or cefepime), more than the currently used protocol, which consists of at least seven therapeutics. Patients experienced more side effects from such currently used multi-drug treatment protocols, and, hence, a simpler treatment protocol proposed in the present study that led to similar outcomes will be better, particularly for elderly patients.

### 3.2. M^Pro^ Inhibitory Activity of the Used Antibiotics

**Figure 2:**
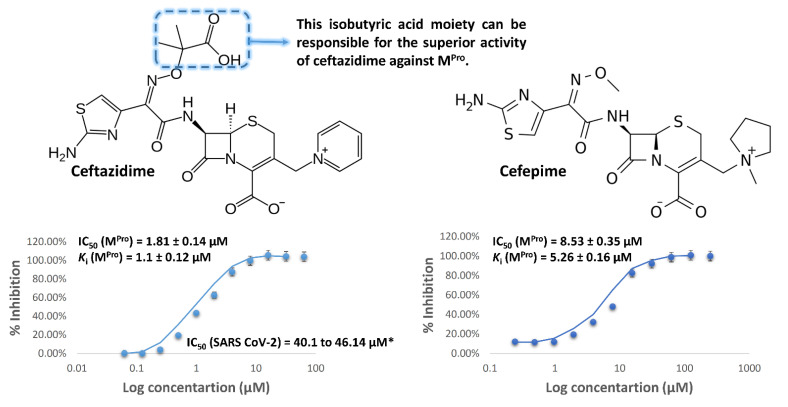
M^Pro^ inhibitory activity of both ceftazidime and cefepime. * Are the previously reported IC_50_ values of ceftazidime against SARS CoV-2 [19].

In vitro testing of both ceftazidime and cefepime against SARS CoV-2′ M^Pro^ (Figure 2) revealed that both of them were able to inhibit the enzyme’s catalytic activity at a micromolar concentration (IC_50_ = 1.81 and 8.53 µM, respectively) with a competitive type of inhibition (*K*_i_ = 1.1 and 5.26 µM, respectively).

However, ceftazidime was significantly more active than cefepime. After careful structural investigation of both compounds, we can conclude that the isobutyric acid moiety of ceftazidime is the main different structural element between the two antibiotics’ scaffolds. Hence, it may contribute to its superior activity against M^Pro^. These results, particularly of ceftazidime, were in perfect accordance and supportive to that of the anti-SARS CoV-2 results (IC_50_ ~ 43 µM) [19].

### 3.3. In-Silico-Based Study of the Mode of Enzyme Inhibition

**Figure 3:**
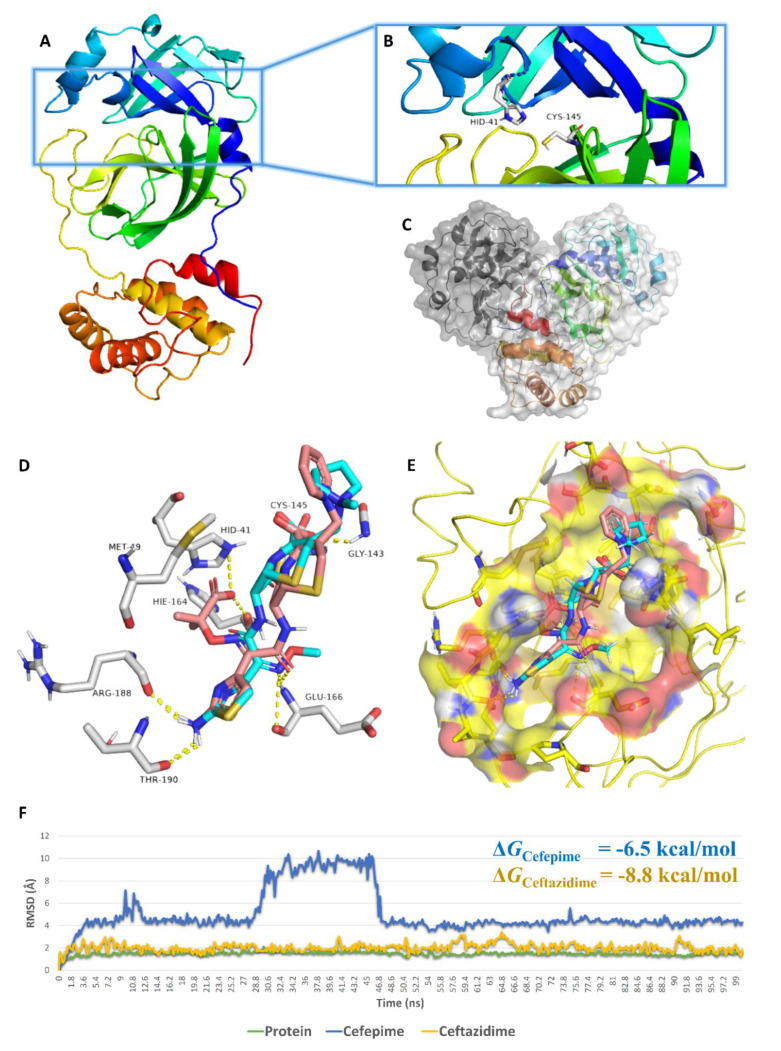
Structure of SARS CoV-2′ M^Pro^ showing its active catalytic site (**A**,**B**) and its dimeric active form (**C**). Binding mode of both ceftazidime and cefepime inside the M^Pro^ active site (**D**,**E**; ceftazidime was colored in brick-red and cyan colors, respectively). RMSDs of both ceftazidime and cefepime inside the M^Pro^ active site during the course of 100 ns fo MDS (**F**).

In order to have a deeper insight into the mode of enzyme inhibition of both antibiotics, we subjected them to in silico molecular docking experiments. We used the enzyme’s catalytic site in these docking experiments, as the in vitro experiments showed that both antibiotics inhibited the enzyme by the competition of the normal substrate for the active site. In our previous work, we demonstrated that this active catalytic site (Figure 3A–C) is flexible; hence, using different molecular dynamic simulation (MDS)-derived conformations of this active site for docking experiments will give more accurate results [1]. Accordingly, we subjected the M^Pro^ crystal structure (PDB: 6LU7) [23] to a 100 ns MDS experiment. Subsequently, we extracted snapshots for the enzyme’s structure every 10 ns. These collected structures were then used for docking experiments, and the average scores of each antibiotic were finally recorded.

All resulting binding modes for both antibiotics were convergent with calculated average docking scores of −7.5 kcal/mol. Therefore, we selected the best-scoring binding mode (i.e., docking score = −7.9 and −7.3 kcal/mol for ceftazidime and cefepime, respectively) for each antibiotic in order to describe their interactions inside the enzyme’s active site. Due to their similar structures, both antibiotics showed the same binding mode inside the active site, and their structures were superimposed onto each other. They were able to establish four strong H-bonds (<2.5 Å) with GLY-143, GLU-166, ARG-188, and THR-190. Additionally, they interacted with both MET-49 and CYS-145 via van der Waals and hydrophobic interactions (Figure 3D,E).

Regarding ceftazidime, it was able to achieve two further important interactions via its extended isobutyric acid moiety. The negatively charged carboxylated group of this moiety was involved in a salt bridge with HID-41 and in an H-bonding with HID-164 (Figure 3D).

Upon further validation of these binding modes by 100 ns MDS experiments, ceftazidime’s binding was obviously more stable (i.e., a lower RMSD over the course of MDS and lower fluctuations) than that of cefepime, where its binding orientation changed dramatically between 27 ns and 46.8 ns (Figure 3E).

**Figure 4:**
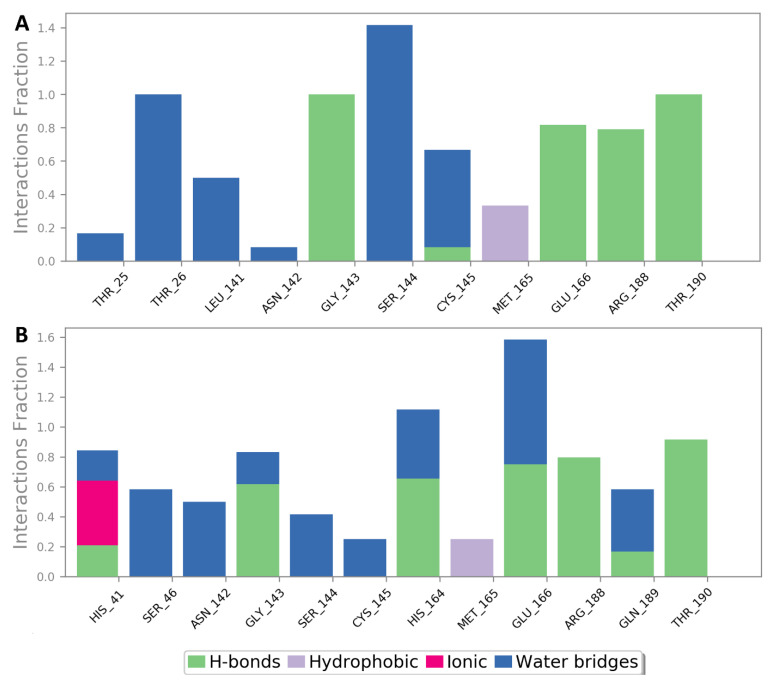
Protein–ligand interactions of both cefepime and ceftazidime (**A**,**B**, respectively) during the course of MDS.

Studying the behavior of both antibiotics during the course of MDS revealed that they were further stabilized inside the enzyme’s active site via a number of water bridges, particularly with SER-144, CYS-145, and THR-26 (Figure 4). Ceftazidime’s extended isobutyric acid moiety contributed to its superior binding stability over cefepime via its stable ionic and H-bonding interactions with HIS-41 and HID-166 throughout the simulation. This higher stability was translated into lower binding free energy (Δ*G* = −8.8 kcal/mol), and it correlated well with its experimental inhibitory results, which showed a superior activity for ceftazidime over cefepime (Figure 2), which obtained a higher Δ*G* value (−6.5 kcal/mol) (Figure 3 and Figure 4).

## 4. Discussion

The third and fourth-generation cephalosporins have been successfully used as empirical antibiotics with hospital-acquired infections. Some of them, such as ceftazidime and cefepime, have shown outstanding outcomes with bacterial pneumonia [31,32].

Herein, we investigated the efficacy of a short-duration glucocorticoid treatment in combination with ceftazidime or cefepime on COVID-19 patients with moderate or severe symptoms, proposing that both antibiotics will also act as antiviral agents besides their protective effect against co-infections and/or superinfections. Generally, the duration of steroid therapy during the treatment of COVID-19 patients ranges from 3 to 12 days [33].

Our results demonstrated that the mean steroid therapy duration needed by the patients who used cefepime or ceftazidime was between 5 to 6 days. This indicates the satisfactory efficacy of cefepime or ceftazidime in treating COVID-19 patients and demonstrates the symptomatic improvement for moderate and severe patients on the 5th or 6th day after starting the treatment. Moreover, when ceftazidime was given in a dose of 1000 mg three times daily for five days, it was noticed that it reduced the duration of symptoms and recovery time in moderate and severe patients with minor adverse effects. Accordingly, this dose regimen can be used to decrease COVID-19 morbidity and mortality.

The previous reported mean duration of COVID-19 symptoms (recovery time) of moderate treated patients was 11.5 days. The symptoms usually vary according to age and sex [34,35].

In this study, the patients used COVID-19 treatments for a period (recovery time) for an average of 10 to 16 days, according to age, which indicates the satisfactory efficacy of cefepime and ceftazidime in the treatment of COVID-19 cases.

The age group (more than 60 years) had a recovery period longer than the age group (31–60 years), and the age group (31–60 years) had a recovery period longer than the age group (18–30 years). This indicates that the recovery time increases with an increasing age. The younger patients have a lower risk of developing into severe cases and recover from COVID-19 symptoms faster than older patients. This is also shown in other studies of Manash. et al. and David Gurwitz [36,37].

The findings showed that using either ceftazidime or cefepime, in combination with steroids, for the treatment of moderate and severe COVID-19 cases resulted in recovery times equivalent to those seen with standard of care treatments (i.e., positive control). As a result, our proposed simple protocol, which consists of only two medications (dexamethasone + either ceftazidime or cefepime), will help patients more than the present complex protocol, which consists of at least seven treatments. Patients had more adverse effects from the current multi-drug therapy regimen, so the proposed simplified treatment plan with good outcomes will, of course, be more preferred, particularly for elderly patients.

On the other hand, both antibiotics showed good inhibitory activity against SARS CoV-2′s M^Pro^ in vitro, where ceftazidime was significantly more active. Further structural and in silico analysis revealed that it could achieve more stable binding with the enzyme’s active site due to its extended isobutyric acid group. The structural and binding mode information provided in this investigation can be extended in the near future to develop more potent non-antibiotic ß-lactam-based SARS CoV-2′s M^Pro^ inhibitors. It is worth noting that ceftazidime has recently been shown to block the interaction between the SARS CoV-2′s spike protein (S-protein) and the human angiotensin-converting enzyme 2 (ACE-2) [32], and, hence, this interesting antibiotic can target multiple targets in SARS CoV-2, providing an excellent scaffold for the development of potent antiviral therapeutics.

The main strengths of our present study are the following: (i) the relatively large sample that was used for the study (370 patients); (ii) the first study to demonstrate the efficacy of ceftazidime and cefepime in the management of severe and moderate COVID-19 cases; (iii) the first study to explore the potential of ceftazidime and cefepime as promising M^pro^ inhibitors; (iv) the outcomes of the study were equivalent to the currently used complex multidrug treatment protocol; (v) the use of in-depth in silico analysis to explore the mode of interaction of these antibiotics with M^pro^.

On the other hand, the main limitations of the present study were (i) its locality (i.e., it was a single-center study), and that (ii) it did not provide conclusive evidence of both antibiotics’ efficacy inside the patient’s body. It was difficult to monitor the viral load in the treated patients in order to come to more comprehensive conclusions on the efficacy of both antibiotics against the virus due to the work overload and exhaustion of hospital resources resulting from the timing of the study.

## 5. Conclusions

Drug repurposing may decrease the time and cost required for developing new drugs. The repurposing of ceftazidime and cefepime is highly effective for the symptomatic improvement of moderate and severe COVID-19 patients. Ceftazidime and cefepime have highly antiviral activity and are effective against various viruses, including SARS and MERS. Dual therapy by combining ceftazidime or cefepime with steroids is considered better than monotherapy with antibiotics or steroids. The recovery time increased with an increasing age. The younger patients have a lower risk of developing into severe cases and recover from COVID-19 symptoms faster than older patients.

According to our findings that ceftazidime or cefepime can currently provide COVID-19 patients extra benefits, being good antiviral agents besides their outstanding antibacterial properties, our main recommendation is to combine these two cephalosporine antibiotics with steroids during the management of moderate and severe COVID-19 cases for better outcomes with minor side effects, instead of the currently used complex multidrug treatment protocol.

## Data Availability

Not applicable.
